# Supplementary material for: Validation of the Micronutrient and Environmental Enteric Dysfunction Assessment Tool and evaluation of biomarker risk factors for growth faltering and vaccine failure in young Malian children
Source: PLoS Negl Trop Dis. 2020 Sep 30;14(9):e0008711. doi: 10.1371/journal.pntd.0008711 (PMC7549819; doi:10.1371/journal.pntd.0008711)
Supplement: S1 Table — (DOCX) [file pntd.0008711.s001.docx]

## S1 Table. Descriptive information, rationale, and previously observed pediatric concentrations for the selected EED or GH axis biomarkers.

| **Marker** | **Full name** | **Indicates** | **Associations/mechanism**  **(rationale)** | **Mean and**  **range* (pg/mL)** |
| --- | --- | --- | --- | --- |
| I-FABP | Intestinal fatty acid–binding protein | Small intestine (enterocyte) injury | •Stunting (cross-sectional)  •Risk of growth faltering | 943  0–16,999 |
| sCD14 | Soluble CD14 | Systemic monocyte activation due to bacterial translocation | •Risk of growth faltering  •Poor immune responses to oral immunizations  •Future cognition scores | 1,949,857  0–18,738,000 |
| FGF21 | Fibroblast growth factor 21 | Growth hormone (GH) resistance due to reduced protein intake | •Risk of growth faltering during nutritional supplementation | 430  25– >2,462 |
| IGF-1 | Insulin-like growth factor 1 | Proper function of the GH axis | •Local binding to receptors promotes tissue and bone growth  •Low IGF-1 suggests GH resistance | 36,796  2,683–84,640 |
| *Estimated from data from pediatric cohorts in Brazil, Bangladesh, and Zimbabwe [1-4]. | | | | |

## References

1. Jiang NM, Tofail F, Ma JZ, Haque R, Kirkpatrick B, Nelson CA, et al. Early Life Inflammation and Neurodevelopmental Outcome in Bangladeshi Infants Growing Up in Adversity. Am J Trop Med Hyg. 2017;97(3):974-9.

2. Naylor C, Lu M, Haque R, Mondal D, Buonomo E, Nayak U, et al. Environmental Enteropathy, Oral Vaccine Failure and Growth Faltering in Infants in Bangladesh. EBioMedicine. 2015;2(11):1759-66.

3. Arndt MB, Richardson BA, Mahfuz M, Ahmed T, Haque R, Gazi MA, et al. Plasma Fibroblast Growth Factor 21 Is Associated with Subsequent Growth in a Cohort of Underweight Children in Bangladesh. Curr Dev Nutr. 2019;3(5):nzz024.

4. Guerrant RL, Leite AM, Pinkerton R, Medeiros PH, Cavalcante PA, DeBoer M, et al. Biomarkers of Environmental Enteropathy, Inflammation, Stunting, and Impaired Growth in Children in Northeast Brazil. PLoS One. 2016;11(9):e0158772.
