# Supplementary material for: Validation of the Micronutrient and Environmental Enteric Dysfunction Assessment Tool and evaluation of biomarker risk factors for growth faltering and vaccine failure in young Malian children
Source: PLoS Negl Trop Dis. 2020 Sep 30;14(9):e0008711. doi: 10.1371/journal.pntd.0008711 (PMC7549819; doi:10.1371/journal.pntd.0008711)
Supplement: S2 Table — (DOCX) [file pntd.0008711.s002.docx]

## S2 Table. Pairwise correlations between biomarkers of environmental enteric dysfunction, the growth hormone axis, systemic inflammation, and micronutrient status.

|  | I-FABP | sCD14 | IGF-1^0^ | FGF21^1^ | AGP | CRP | GLP-2^2^ | Ferritin | RBP4 | sTfR |
| --- | --- | --- | --- | --- | --- | --- | --- | --- | --- | --- |
| I-FABP | 1 |  |  |  |  |  |  |  |  |  |
| sCD14 | 0.1887^*^ | 1 |  |  |  |  |  |  |  |  |
| IGF-1^0^ | −0.0346 | 0.2196^†^ | 1 |  |  |  |  |  |  |  |
| FGF21^1^ | 0.1034 | 0.2054^*^ | −0.0871 | 1 |  |  |  |  |  |  |
| AGP | 0.2527^‡^ | 0.3463^‡^ | −0.1691^*^ | 0.3550^‡^ | 1 |  |  |  |  |  |
| CRP | 0.0280 | 0.1570^*^ | −0.1705^*^ | 0.1700^*^ | 0.5456^‡^ | 1 |  |  |  |  |
| GLP-2^2^ | 0.2863^†^ | 0.0620 | −0.1172 | 0.1598 | −0.0087 | −0.0966 | 1 |  |  |  |
| Ferritin | 0.5241^‡^ | 0.3148^‡^ | −0.0888 | 0.2186^†^ | 0.3535^‡^ | 0.2217^†^ | 0.0673 | 1 |  |  |
| RBP4 | 0.1009 | 0.3786^‡^ | 0.2397^†^ | −0.0006 | −0.0188 | −0.1808^*^ | 0.1214 | 0.1274 | 1 |  |
| sTfR | −0.0228 | 0.0148 | −0.0319 | 0.0277 | 0.1134^*^ | 0.0233 | 0.0770 | −0.1283^*^ | −0.0405 | 1 |

*Abbreviations:* AGP, α1-acid glycoprotein; CRP, C-reactive protein; FGF21, fibroblast growth factor 21; GLP-2, glucagon-like peptide 2; I-FABP, intestinal fatty acid–binding protein; IGF-1, insulin-like growth factor 1; RBP4, retinol binding protein-4; sCD14, soluble cluster of differentiation 14; sTfR, soluble transferrin receptor.

^0^ n = 258 for IGF-1 correlation coefficients with the exception of FGF21 (n = 256) and GLP-2 (n = 124), as values below the LLOQ (2.7 ng/ml) are excluded.

^1^ n = 296 for FGF21 correlation coefficients with the exception of IGF-1 (n = 256) and GLP-2 (n = 150), as values below the LLOQ (10 pg/ml) are excluded.

^2^ n = 152 for GLP-2 correlation coefficients with the exception of IGF-1 (n = 124) and GLP-2 (n = 150), as GLP-2 was tested only in a subset of 152 children.

∗, **†, ‡** Statistically significant: ∗ P < 0.05, **†** P < 0.001, **‡** P < 0.0001.
