# Supplementary material for: Validation of the Micronutrient and Environmental Enteric Dysfunction Assessment Tool and evaluation of biomarker risk factors for growth faltering and vaccine failure in young Malian children
Source: PLoS Negl Trop Dis. 2020 Sep 30;14(9):e0008711. doi: 10.1371/journal.pntd.0008711 (PMC7549819; doi:10.1371/journal.pntd.0008711)
Supplement: S3 Table — (DOCX) [file pntd.0008711.s003.docx]

## S3 Table. Rotavirus vaccine seroconversion at 28 days post-immunization by EED and GH status measured by ELISA, in infants without natural rotavirus infection during the 28 days of follow-up (n = 220 unless otherwise noted).

| **Biomarker and quartile** | **No. of infants**  **(% of total)** | **No. (%) seroconverted (IgA ≥**  **threefold increase)** | **Trend test**  **P-value** | **No. (%) seroconverted (IgG ≥ threefold increase)** | **Trend test**  **P-value** |
| --- | --- | --- | --- | --- | --- |
| **I-FABP—quartiles (cutoffs in pg/mL)** | | | | | |
| **1** (< 504.2) | 46 (20.9) | 23 (50.0) | 0.137 | 27 (58.7) | **0.034** |
| **2** (< 820.4) | 59 (26.8) | 36 (61.0) |  | 42 (71.2) |  |
| **3** (< 1200.3) | 58 (26.4) | 28 (48.3) |  | 29 (50.0) |  |
| **4** (> 1200.3) | 57 (25.9) | 23 (40.4) |  | 26 (45.6) |  |
| **sCD14—quartiles (cutoffs in ng/mL)** | | | | | |
| **1** (< 1297.2) | 50 (22.9) | 28 (56.0) | 0.091 | 33 (66.0) | **0.036** |
| **2** (< 1615.7) | 50 (22.9) | 29 (58.0) |  | 30 (60.0) |  |
| **3** (< 1867.2) | 58 (26.6) | 26 (44.8) |  | 32 (55.2) |  |
| **4** (> 1867.2) | 60 (27.5) | 26 (43.3) |  | 28 (46.7) |  |
| **IGF-1—quartiles (cutoffs in ng/mL)** | | | | | |
| **1** (< 12.2) | 56 (25.5) | 31 (55.4) | 0.286 | 35 (62.5) | 0.905 |
| **2** (< 19.7) | 56 (25.5) | 33 (58.9) |  | 31 (55.4) |  |
| **3** (< 27.8) | 50 (22.7) | 15 (30.0) |  | 20 (40.0) |  |
| **4** (> 27.8) | 58 (26.4) | 31 (53.5) |  | 38 (65.5) |  |
| **FGF21—quartiles (cutoffs in pg/mL)** | | | | | |
| **1** (< 92.4) | 49 (22.3) | 28 (57.1) | **0.035** | 29 (59.2) | 0.606 |
| **2** (< 163.6) | 48 (21.8) | 28 (58.3) |  | 29 (60.4) |  |
| **3** (< 345.7) | 60 (27.3) | 29 (48.3) |  | 35 (58.3) |  |
| **4** (> 345.7) | 63 (28.6) | 25 (39.7) |  | 31 (49.2) |  |

*Abbreviations:* CI, confidence interval; CRP, FGF21, fibroblast growth factor 21; I-FABP, intestinal fatty acid–binding protein; IGF-1, insulin-like growth factor 1; IgA, immunoglobulin A; IgG, immunoglobulin G; sCD14, soluble cluster of differentiation 14.

^0^ n = 299 for IGF-1.
