# Supplementary material for: Validation of the Micronutrient and Environmental Enteric Dysfunction Assessment Tool and evaluation of biomarker risk factors for growth faltering and vaccine failure in young Malian children
Source: PLoS Negl Trop Dis. 2020 Sep 30;14(9):e0008711. doi: 10.1371/journal.pntd.0008711 (PMC7549819; doi:10.1371/journal.pntd.0008711)
Supplement: S4 Table — (DOCX) [file pntd.0008711.s004.docx]

## S4 Table. Yellow fever and meningococcal A vaccine seroconversion at 28 days post-immunization by EED and GH status measured by ELISA (N = 300 unless otherwise noted).

| **Biomarker and quartile** | **No. of infants**  **(% of total)** | **No. (%) seroconverted to YFV (NT ≥ fourfold increase)** | **Trend test**  **P-value** | **No. (%) seroconverted to MenAV (SBA ≥ fourfold increase)** | **Trend test**  **P-value** |
| --- | --- | --- | --- | --- | --- |
| **I-FABP—quartiles (cutoffs in pg/mL)** | | | | | |
| **1** (< 504.2) | 75 (25.0) | 49 (65.3) | 0.224 | 69 (92) | 1.000 |
| **2** (< 820.4) | 75 (25.0) | 51 (68.0) |  | 74 (98.67) |  |
| **3** (< 1200.3) | 75 (25.0) | 46 (61.3) |  | 71 (94.67) |  |
| **4** (> 1200.3) | 75 (25.0) | 58 (77.3) |  | 70 (93.33) |  |
| **sCD14—quartiles (cutoffs in ng/mL)** | | | | | |
| **1** (< 1297.2) | 75 (25.2) | 49 (65.3) | 0.724 | 73 (97.33) | 0.352 |
| **2** (< 1615.7) | 74 (24.8) | 52 (70.3) |  | 69 (93.24) |  |
| **3** (< 1867.2) | 75 (25.2) | 51 (68.0) |  | 71 (94.67) |  |
| **4** (> 1867.2) | 74 (24.8) | 51 (68.9) |  | 69 (93.24) |  |
| **IGF-1—quartiles (cutoffs in ng/mL)^0^** | | | | | |
| **1** (< 12.2) | 76 (25.3) | 41 (54.0) | **0.002** | 72 (94.74) | 0.461 |
| **2** (< 19.7) | 77 (25.3) | 55 (72.4) |  | 74 (97.37) |  |
| **3** (< 27.8) | 74 (24.7) | 48 (64.9) |  | 69 (93.24) |  |
| **4** (> 27.8) | 75 (24.7) | 60 (81.1) |  | 69 (93.24) |  |
| **FGF21—quartiles (cutoffs in pg/mL)** | | | | | |
| **1** (< 92.4) | 75 (25.0) | 49 (65.3) | 0.912 | 70 (93.33) | 0.646 |
| **2** (< 163.6) | 75 (25.0) | 54 (72.0) |  | 73 (97.33) |  |
| **3** (< 345.7) | 75 (25.0) | 50 (66.7) |  | 72 (96) |  |
| **4** (> 345.7) | 75 (25.0) | 51 (68.0) |  | 69 (92) |  |

*Abbreviations:* CI, confidence interval; FGF21, fibroblast growth factor 21; I-FABP, intestinal fatty acid–binding protein; IGF-1, insulin-like growth factor 1; NT, neutralizing antibody titer; SBA, serum bactericidal assay; sCD14, soluble cluster of differentiation 14.

^0^ n = 298 for sCD14.

^1^ n = 152 for GLP-2.
