# Supplementary material for: Validation of the Micronutrient and Environmental Enteric Dysfunction Assessment Tool and evaluation of biomarker risk factors for growth faltering and vaccine failure in young Malian children
Source: PLoS Negl Trop Dis. 2020 Sep 30;14(9):e0008711. doi: 10.1371/journal.pntd.0008711 (PMC7549819; doi:10.1371/journal.pntd.0008711)
Supplement: S5 Table — (DOCX) [file pntd.0008711.s005.docx]

## S5 Table. Associations between baseline log-2 transformed serum biomarker concentrations (ELISA) with 28-day seroconversion to rotavirus, yellow fever, and meningococcal A vaccines.

|  |  | **Crude RR** | **(95% CI)** | **P-value** | **Adjusted RR^0^** | **(95% CI)** | **P-value** |
| --- | --- | --- | --- | --- | --- | --- | --- |
| **Rotavirus seroconversion (IgA ≥ threefold increase)^1^** | I-FABP | 0.97 | (0.86, 1.10) | 0.651 | 1.02 | (0.92, 1.14) | 0.665 |
|  | sCD14 | 0.82 | (0.70, 0.97) | **0.018** | 0.97 | (0.82, 1.14) | 0.694 |
|  | IGF-1 | 0.91 | (0.83, 1.00) | 0.062 | 0.91 | (0.84, 0.99) | **0.036** |
|  | FGF21 | 0.91 | (0.84, 0.99) | **0.035** | 0.93 | (0.86, 1.01) | 0.080 |
|  | AGP | 0.96 | (0.78, 1.17) | 0.680 | 1.06 | (0.84, 1.35) | 0.598 |
|  | CRP | 0.98 | (0.93, 1.03) | 0.400 | 0.97 | (0.91, 1.04) | 0.421 |
|  | GLP-2^3^ | 1.05 | (0.82, 1.36) | 0.691 | 1.08 | (0.83, 1.39) | 0.564 |
|  |  |  |  |  |  |  |  |
| **Rotavirus seroconversion (IgG ≥ threefold increase)^1^** | I-FABP | 0.91 | (0.82, 1.01) | 0.072 | 0.94 | (0.85, 1.03) | 0.189 |
|  | sCD14 | 0.91 | (0.72, 1.14) | 0.403 | 1.07 | (0.84, 1.36) | 0.574 |
|  | IGF-1 | 0.97 | (0.88, 1.06) | 0.502 | 0.96 | (0.89, 1.04) | 0.353 |
|  | FGF21 | 0.95 | (0.88, 1.03) | 0.192 | 0.96 | (0.89, 1.04) | 0.312 |
|  | AGP | 0.95 | (0.78, 1.15) | 0.596 | 1.05 | (0.84, 1.33) | 0.657 |
|  | CRP | 0.98 | (0.93, 1.02) | 0.331 | 0.98 | (0.92, 1.04) | 0.485 |
|  | GLP-2^3^ | 0.97 | (0.79, 1.20) | 0.782 | 1.03 | (0.83, 1.28) | 0.786 |
|  |  |  |  |  |  |  |  |
| **Yellow fever seroconversion (NT ≥ fourfold increase)^2^** | I-FABP | 1.03 | (0.96, 1.09) | 0.452 | 1.01 | (0.95, 1.08) | 0.65 |
|  | sCD14 | 1.02 | (0.88, 1.18) | 0.781 | 1.02 | (0.89, 1.18) | 0.771 |
|  | IGF-1 | 1.13 | (1.04, 1.23) | **0.005** | 1.06 | (0.99, 1.14) | 0.115 |
|  | FGF21 | 1.02 | (0.97, 1.07) | 0.485 | 1.01 | (0.96, 1.05) | 0.807 |
|  | AGP | 0.94 | (0.86, 1.03) | 0.158 | 0.92 | (0.84, 1.01) | 0.075 |
|  | CRP | 1.01 | (0.97, 1.04) | 0.752 | 1.02 | (0.98, 1.06) | 0.414 |
|  | GLP-2^4^ | 1.11 | (0.90, 1.36) | 0.337 | 1.13 | (0.93, 1.36) | 0.224 |
|  |  |  |  |  |  |  |  |
| **Meningococcus A seroconversion (SBA ≥ fourfold increase)^2^** | I-FABP | 0.98 | (0.96, 1.01) | 0.281 | 0.99 | (0.96, 1.02) | 0.435 |
|  | sCD14 | 0.98 | (0.93, 1.02) | 0.287 | 0.99 | (0.95, 1.03) | 0.515 |
|  | IGF-1 | 0.99 | (0.97, 1.01) | 0.431 | 0.99 | (0.97, 1.01) | 0.356 |
|  | FGF21 | 0.99 | (0.97, 1.02) | 0.529 | 1.00 | (0.98, 1.02) | 0.740 |
|  | AGP | 0.97 | (0.94, 1.00) | 0.066 | 0.98 | (0.95, 1.01) | 0.219 |
|  | CRP | 1.00 | (0.98, 1.01) | 0.395 | 1.00 | (0.99, 1.01) | 0.745 |
|  | GLP-2^4^ | 0.97 | (0.92, 1.03) | 0.319 | 0.98 | (0.93, 1.03) | 0.408 |

*Abbreviations:* AGP, α1-acid glycoprotein; CI, confidence interval; CRP, C-reactive protein; FGF21, fibroblast growth factor 21; GLP-2, glucagon-like peptide 2; I-FABP, intestinal fatty acid–binding protein; IGF-1, insulin-like growth factor 1; IgA, immunoglobulin A; IgG, immunoglobulin G; NT, neutralizing antibody titer; RR, relative risk; SBA, serum bactericidal assay; sCD14, soluble cluster of differentiation 14.

^0^ Estimates were adjusted for other log-transformed biomarkers (except GLP-2), age of infant (in months) at vaccination, relevant baseline log-transformed immunological titer(s).

^1^ n = 220 children for rotavirus seroconversion analyses: excluding those with increased titer who reported diarrhea, gastroenteritis, or vomiting in the period; n = 218 for sCD14 univariable and all adjusted RR (excluding GLP-2).

^2^ n = 300; n = 298 for sCD14 univariable and adjusted models (excluding GLP-2).

^3^ n = 133 for GLP-2 univariable RR for rotavirus seroconversion.

^4^ n = 152 for GLP-2 univariable RR for YFV and MenAV seroconversion.
