# Supplementary material for: Validation of the Micronutrient and Environmental Enteric Dysfunction Assessment Tool and evaluation of biomarker risk factors for growth faltering and vaccine failure in young Malian children
Source: PLoS Negl Trop Dis. 2020 Sep 30;14(9):e0008711. doi: 10.1371/journal.pntd.0008711 (PMC7549819; doi:10.1371/journal.pntd.0008711)
Supplement: S6 Table — (DOCX) [file pntd.0008711.s006.docx]

## S6 Table. Associations between baseline log-2 transformed serum biomarker concentrations (ELISA) with change in HAZ, WAZ, and WHZ over 12 weeks of follow up.

|  |  | **Crude coefficient** | **(95% CI)** | **P−value** | **Adjusted coefficient^0^** | **(95% CI)** | **P−value** |
| --- | --- | --- | --- | --- | --- | --- | --- |
| **ΔHAZ** | I-FABP | 0.05 | (0.00, 0.10) | **0.042** | 0.04 | (−0.01, 0.09) | 0.089 |
|  | sCD14 | 0.01 | (−0.09, 0.11) | 0.797 | 0.03 | (−0.10, 0.15) | 0.691 |
|  | IGF-1**^1^** | −0.05 | (−0.11, 0.01) | 0.076 | −0.03 | (−0.08, 0.02) | 0.290 |
|  | FGF21 | −0.01 | (−0.04, 0.02) | 0.605 | −0.03 | (−0.07, 0.00) | 0.073 |
|  | AGP | 0.08 | (−0.01, 0.17) | 0.098 | 0.08 | (−0.04, 0.21) | 0.176 |
|  | CRP | 0.01 | (−0.02, 0.03) | 0.626 | −0.02 | (−0.05, 0.01) | 0.263 |
|  | GLP-2^2^ | 0.11 | (−0.03, 0.24) | 0.132 | 1.03 | (0.83, 1.28) | 0.786 |
|  | Ferritin | 0.01 | (−0.02, 0.03) | 0.706 | 0.00 | (−0.02, 0.03) | 0.842 |
|  | RBP4 | −0.01 | (−0.17, 0.14) | 0.860 | 0.01 | (−0.13, 0.14) | 0.936 |
|  |  |  |  |  |  |  |  |
| **ΔWAZ** | I-FABP | 0.02 | (−0.04, 0.07) | 0.553 | 0.02 | (−0.06, 0.05) | 0.776 |
|  | sCD14 | −0.08 | (−0.21, 0.04) | 0.184 | −0.08 | (−0.28, −0.01) | **0.032** |
|  | IGF-1**^1^** | −0.04 | (−0.11, 0.02) | 0.173 | −0.04 | (−0.07, 0.06) | 0.916 |
|  | FGF21 | 0.04 | (−0.01, 0.08) | 0.136 | 0.04 | (−0.02, 0.08) | 0.282 |
|  | AGP | 0.09 | (−0.02, 0.19) | 0.116 | 0.06 | (−0.10, 0.22) | 0.471 |
|  | CRP | 0.01 | (−0.01, 0.04) | 0.339 | 0.00 | (−0.04, 0.04) | 0.939 |
|  | GLP-2^2^ | −0.15 | (−0.29, −0.01) | **0.037** | −0.19 | (−0.32, −0.06) | **0.005** |
|  | Ferritin | 0.04 | (0.00, 0.07) | **0.029** | 0.03 | (0.00, 0.07) | 0.061 |
|  | RBP4 | 0.03 | (−0.09, 0.16) | 0.592 | 0.03 | (−0.14, 0.20) | 0.717 |
|  |  |  |  |  |  |  |  |
| **ΔWHZ** | I-FABP | 0.02 | (−0.09, 0.07) | 0.758 | 0.02 | (−0.13, 0.03) | 0.228 |
|  | sCD14 | −0.08 | (−0.29, 0.06) | 0.200 | −0.08 | (−0.38, −0.03) | **0.023** |
|  | IGF-1**^1^** | −0.04 | (−0.10, 0.08) | 0.773 | −0.04 | (−0.04, 0.15) | 0.251 |
|  | FGF21 | 0.04 | (−0.01, 0.12) | 0.101 | 0.04 | (−0.02, 0.12) | 0.129 |
|  | AGP | 0.07 | (−0.10, 0.23) | 0.435 | 0.08 | (−0.14, 0.30) | 0.463 |
|  | CRP | 0.01 | (−0.03, 0.05) | 0.532 | 0.01 | (−0.05, 0.06) | 0.810 |
|  | GLP-2^2^ | −0.29 | (−0.50, −0.08) | **0.007** | −0.30 | (−0.51, −0.10) | **0.005** |
|  | Ferritin | 0.04 | (−0.01, 0.09) | 0.095 | 0.03 | (−0.01, 0.08) | 0.164 |
|  | RBP4 | −0.01 | (−0.20, 0.18) | 0.884 | −0.02 | (−0.25, 0.22) | 0.892 |

*Abbreviations:* AGP, α1-acid glycoprotein; CI, confidence interval; CRP, C-reactive protein; FGF21, fibroblast growth factor 21; GLP-2, glucagon-like peptide 2; I-FABP, intestinal fatty acid–binding protein; IGF-1, insulin-like growth factor 1; RBP4, retinol binding protein-4; sCD14, soluble cluster of differentiation 14.

^0^ Estimates were adjusted for other log-transformed biomarkers (except for GLP-2), sex, days of diarrhea in the first 28 days, relevant baseline anthropometric measurement (i.e., HAZ, WAZ, or WHZ).

^1^ n = 299 children with valid anthropometric measurements at day 84 of follow up; n = 297 for sCD14 univariable and all adjusted models (excluding GLP-2).

^2^ n = 151 who were tested for GLP-2 with valid anthropometric measurements at day 84 of follow-up are featured in crude and adjusted models for GLP-2.
