# Supplementary material for: Validation of the Micronutrient and Environmental Enteric Dysfunction Assessment Tool and evaluation of biomarker risk factors for growth faltering and vaccine failure in young Malian children
Source: PLoS Negl Trop Dis. 2020 Sep 30;14(9):e0008711. doi: 10.1371/journal.pntd.0008711 (PMC7549819; doi:10.1371/journal.pntd.0008711)
Supplement: S7 Table — (DOCX) [file pntd.0008711.s007.docx]

## S7 Table. Comparison of baseline serum biomarker concentrations (ELISA) in children with vs. without putative rotavirus infection in 28 days of follow-up.

|  | **Infected^0^ (n = 27)** | | **Uninfected^1^ (n = 27)** | |  |
| --- | --- | --- | --- | --- | --- |
|  | **mean** | **(95% CI)** | **mean** | **(95% CI)** | **T-test^2^ P-value** |
| I-FABP (pg/mL) | 1485.0 | (365.4, 2604.6) | 873.4 | (630.8, 1116.1) | 0.2817 |
| sCD14 (ng/mL) | 1488.86 | (1233.35, 1744.37) | 1381.60 | (1206.33, 1556.86) | 0.4803 |
| IGF-1 (ng/mL) | 20.0 | (15.5, 24.5) | 21.0 | (16.6, 25.4) | 0.7531 |
| FGF21 (pg/mL) | 182.94 | (105.74, 260.15) | 221.40 | (111.03, 331.77) | 0.5601 |
|  |  |  |  |  |  |

*Abbreviations:* AGP, α1-acid glycoprotein; CI, confidence interval; CRP, C-reactive protein; FGF21, fibroblast growth factor 21; I-FABP, intestinal fatty acid–binding protein; IGF-1, insulin-like growth factor 1; IgA, immunoglobulin A; IgG, immunoglobulin G; RBP4, retinol binding protein-4; sCD14, soluble cluster of differentiation 14.

^0^ Infants in the non-PRV arm with both anti-rotavirus IgG < 20 units/mL and IgA < 20 units/mL at enrollment, with ≥ threefold rise in IgG and/or IgA after 28 days of follow-up.

^1^ Infants in the non-PRV arm with both anti-rotavirus IgG < 20 units/mL and IgA < 20 units/mL at enrollment, with < threefold rise in both IgG and IgA after 28 days of follow-up.

^2^Two-sample Student’s t-test with unequal variances.
