# Supplementary material for: Validation of the Micronutrient and Environmental Enteric Dysfunction Assessment Tool and evaluation of biomarker risk factors for growth faltering and vaccine failure in young Malian children
Source: PLoS Negl Trop Dis. 2020 Sep 30;14(9):e0008711. doi: 10.1371/journal.pntd.0008711 (PMC7549819; doi:10.1371/journal.pntd.0008711)
Supplement: Attachment — Submitted filename: PNTD_MEEDAT_Review_responses20200303.docx [file pntd.0008711.s010.docx]

# Authors’ responses to Reviewer Comments

Key Review Criteria Required for Acceptance?

**Reviewer #1: The manuscript focuses on associations between four different outcomes and multiple biomarkers, each measured in two different ways (MEEDAT versus ELISA) and using two different methods of statistical analysis. It is therefore very hard to take in all these findings. Furthermore, the findings are generally over-interpreted with too much emphasis on P values rather than effect sizes with an accompanying measure of precision, and equal weight given in interpretation to univariable and multivariable analysis. There is a general lack of hypotheses throughout; for example, it is unclear why the authors would evaluate associations between IGF-1 and yellow fever vaccine seroconversion: what is the hypothesis underlying this question?**

We thank reviewer #1 for their thoughtful review, and acknowledge that the original draft placed too much emphasis on p-values and did not distinguish adequately between the interpretation of crude and adjusted estimates of the associations. We have revised the manuscript results and abstract to mitigate these issues and have placed more emphasis on the estimate(s) effect size, as suggested. We have also added some text to explain that some of the analyses conducted were strictly exploratory in nature. For example, the evaluation of the association between IGF-1 (and other MEEDAT biomarkers) with systemic vaccine seroconversion was intended to draw contrast with the analyses where our hypotheses were grounded in the existing evidence regarding systemic inflammation, gut dysfunction, and immune responses to oral vaccines. We have revised the results and conclusions sections to emphasize the exploratory nature of such analyses and downplay the importance of crude associations that were not significant following adjustment in multivariable analysis. For example, the following text was added to the introduction section:

Although prior studies have observed relationships between immune response to orally administered vaccinations with EED and systemic inflammation biomarkers, few studies to date have tested whether biomarkers of GH resistance, systemic inflammation, or EED are associated with responses to parenteral vaccines [12]. No studies have tested if such biomarkers are associated with risk of natural rotavirus infection. A priori, we expect that EED would not be associated with parenteral vaccine response due to the lack of mucosal immune system involvement.

Reviewer #2: 1. Are the objectives of the study clearly articulated with a clear testable hypothesis stated?

**Adding a line clearly defined the objectives of this work is missing and should ideally be placed at the end of introduction**

We are grateful for this critical feedback and have added several lines to the background/introduction section that clearly define the study objectives:

The primary study objective was to test the laboratory performance of the novel multiplex assay by using it to quantitate plasma concentrations of IGF-1, FGF21, sCD14, I-FABP in child specimens and then compare with concentrations measured by monoplex ELISAs. The secondary objectives were to test if biomarkers of EED, systemic inflammation, and growth hormone resistance at baseline were associated with seroconversion to oral rotavirus vaccination and 12-week height/weight growth. Finally, the exploratory objectives were to test if these biomarkers were associated with 1) seroconversion to parenteral meningococcus A or yellow fever vaccines and 2) natural rotavirus infection in children not given PRV.

2. -Is the study design appropriate to address the stated objectives?

**Flow chart for study design is missing**

We have added a study design flow chart (Fig 2) that describes the study population used to address the various objectives. We have also chosen to remove the biorepository specimen data and background from the manuscript as it seems that their inclusion was causing confusion for reviewers and manuscript clarity and accessibility outweigh the scientific benefit(s) of including those preliminary observations.

3. Is the population clearly described and appropriate for the hypothesis being tested?

**yes, but a supplementary table would help clearly identifying group with numbers in each sub analyses group, they have used different numbers for sub analyses**

We thank the reviewer for this important feedback and have added a study design flow chart that helps to more clearly identify groups in each sub analyses group. This was an oversight in the original manuscript.

4. Is the sample size sufficient to ensure adequate power to address the hypothesis being tested?

**yes adequate, though sample size calculation is not given**

We thank the reviewer for this feedback. Sample sizes were driven by sample availability from non-PRV group children who had a rise in anti-rota immunoglobin titer. However, we have added the following text to the limitations section to address the sample size.

Case-control sample sizes were driven by sample availability from non-PRV recipients who had a rise in anti-rotavirus IgA or IgG over 28 days, and this sample size may not have provided adequate power to detect a statistically significant difference in baseline biomarker concentrations. Future work to answer this important question will need to come from a larger (preferably prospective) cohort of non-immunized children.

5. -Were correct statistical analysis used to support conclusions?

**yes- suggest to get it reviewed by a Biostatistician- I cannot make comments on Regression on Log2 transformation, if it is appropriate?**

Regression with Log2 transformed data is a well-accepted approach for the analysis of biomarker concentrations.  See for example:

Mwape, I., S. Bosomprah, J. Mwaba, K. Mwila-Kazimbaya, N. M. Laban, C. C. Chisenga, G. Sijumbila, M. Simuyandi and R. Chilengi (2017). "Immunogenicity of rotavirus vaccine (RotarixTM) in infants with environmental enteric dysfunction." PLoS One 12(12): e0187761.

6. Are there concerns about ethical or regulatory requirements being met?

all ethical approval are taken. in case of bio-repository specimen, was ethical exemption obtained?

**Reviewer #3: The Methods appeared adequate.**

--------------------

Results

-Does the analysis presented match the analysis plan?

-Are the results clearly and completely presented?

-Are the figures (Tables, Images) of sufficient quality for clarity?

Reviewer #1: See above

Reviewer #2: Does the analysis presented match the analysis plan?

**it is adequate but a reviewer or statistician should review it for its correct interpretation. This is beyond my expertise. however, authors has not mentioned if p values are FDR corrected?**

We thank the reviewer for this feedback.  We have added the following text to the limitations section to address are choice not to conduct any correction for multiple comparisons for our exploratory analyses:

Although numerous statistical tests are run and results presented in the study (e.g., Tables 3-6), no formal effort was made to control for the family-wise error rate such as the use of the Benjamini–Hochberg or Bonferroni method for alpha-correction. However, the use of unadjusted P-values is acceptable in exploratory analyses such as those in this study.

-Are the results clearly and completely presented?

**Results are presented clearly. I would suggest to add a figure of Meedat platform, a pictorial will be helpful for a regular panel and analytes that were used in this study. I donot understand mentioning of micro-nutrient panel in abstract as these results were not shown in the manuscript at all. Please clarify if these analytes were tested on these samples and results of only 4 GH resistance markers are described in this paper? it seems that there are 18 analytes per well.**

A pictorial of the MEEDAT platform has been added to the methods section of the manuscript as Figure 1. The micronutrient panel was mentioned in the abstract to make clear that this panel is an expansion of an existing commercial product. These analytes were tested on these samples, however only the results of AGP, CRP, IGF-1, sCD14, I-FABP, and FGF21 are described in this paper. There are a total of 11 analytes per well, as can be seen on the new pictorial.

-Are the figures (Tables, Images) of s ufficient quality for clarity?

**titles of tables are quite long, also figure legend is missing for figure 1 and 2.**

We thank the reviewer for this feedback. All table titles have been shortened and Fig 1 is now Fig 3. The legend for Fig 3 is along the bottom of the figure, above the Abbreviations.

**table S3 and S4 are ELISA based results but title of table 4 and 5 are very much similar to suppl tables. please specify if table 4 and 5 are only MEEDAT results ? why suppl. tables have less biomarkers tested by ELISA? it would be better to mention table # in results and in discussion section. Reader seem to get easily lost as what analyses author is referring to?**

We thank the reviewer for their comment. The Tables 4 and 5 in the original manuscript are now titled Tables 3 and 4 due to the removal of biorepository data, and the text “measured by MEEDAT” has been added to the titles to make clear the source of the biomarker data. The supplemental tables have less biomarkers tested by ELISA because we did not test for AGP or CRP with mono-plex ELISA due to the previous performance testing that has been conducted on the existing 7-plex assay. Table numbers have been added to the discussion section, per the reviewer recommendation.

**Abstract: line 34, WHZ instead of weight for height**

This sentence in the abstract has been revised to include “z-score”.

**line 19: micronutrient deficiency can be removed**

This sentence in the abstract has been revised per this comment.

**line124, pg 7: 1 biomarker can be changed to single biomarker**

Updated, these comments were quite helpful.

**Method: line 174, please give "n" for children excluded due to natural rota virus infection**

We apologize for this oversight, and have now incorporated into a study flow diagram the n=26 children who were excluded due to natural rota infection. These are mentioned explicitly in the first paragraph of the results section.

**line 230 and 231, pg 11: mention the name of labs where NT and SBA were performed.**

These details have been added to the methods section:

Yellow fever virus–specific neutralizing antibody titer (NT) was determined at the Robert Koch Institute (Berlin, Germany) using the Institute’s methods [28]. A validated serum bactericidal assay (SBA) that uses infant rabbit complement was used to measure the titer of functional antibody in human sera to Neisseria meningitidis group A in the Vaccine Evaluation Unit laboratory at Public Health England (Manchester, UK).

**line 306, page 15: please add "using a Poisson working .."**

Revised.

**line 307, pg 15: "are"**

Revised.

**table 3: Only female (47.7%) are give in this table. Is it assumed that 52.3% are male. Why male was not mentioned here?**

For concision, only the percent female is given, so it can be assumed that 52.3% are male.

**line 543, pg 29: WFH, is it WHZ?**

We thank the reviewer for their attention to detail, indeed the WFH was supposed to be WHZ. This sentence has been revised accordingly.

**I am wondering whether MEEDAT and ELISA median levels were compared by MWU test? please add column for p value. Anthropometric comparison for enrollment and 12 weeks can be done parallel to each other characteristics should not be repeated.**

We thank the reviewer for this query, however we do not believe that testing if the central tendency of these two tests (e.g., the median values) were identical for each of the 4 new MEEDAT biomarkers adds meaningfully to the analysis and interpretation. Within a study, the biomarker quantification method must be valid and used consistently. Thus we are not concerned about systematic differences between the average values from the two methods. The study was interested in intra-method comparisons on the individual level, and these results have been provided in the text. Therefore, we feel it is more informative to focus on the interpretation of intra-method comparisons rather than including additional exploratory p-values.

**Reviewer #3: The Results appeared adequate.**

--------------------

Conclusions

-Are the conclusions supported by the data presented?

-Are the limitations of analysis clearly described?

-Do the authors discuss how these data can be helpful to advance our understanding of the topic under study?

-Is public health relevance addressed?

**Reviewer #1: See above**

**Reviewer #2: -Are the conclusions supported by the data presented?**

**yes field applicability of MEEDAT should be discussed**

We appreciate this vital feedback from the reviewer and have added some text to the strengths section about the field applicability of MEEDAT.

**-Are the limitations of analysis clearly described?**

**yes adequately described**

-Do the authors discuss how these data can be helpful to advance our understanding of the topic under study?

**yes, adequate discussion in conclusion**

-Is public health relevance addressed?

**Application of technology in terms of field application needs to be discussed such as a standard microbiology lab equipped with standard equipment can perform and interpret this test. Does this test require esp equipment, esp operational needs, training os staff etc?.**

We appreciate this vital feedback from the reviewer and have added considerable text to the strengths section about the field applicability of MEEDAT:

Another strength of this study was the use of a technology suitable for deployment in low-resource setting laboratories to quantitate biomarkers in serum samples with MEEDAT. Use of the Quansys Q-View™ Imager LS requires a standard wet (e.g., microbiology) laboratory, minimal training of technicians familiar with running ELISA protocols, and a basic laptop computer for data processing and reports. The Imager LS platform is particularly well-suited for use in low-resource settings due to its comparatively low cost, few moving parts, small footprint, and protocol similarity to standard ELISA.

**Discussion is too long. please summarize important finding with support of data. I suggest a way to present comparison of MEEDAT data in the first part and seroconversion in the second part.**

We have attempted to balance the need for a comprehensive discussion of the study findings with a desire for concision. We hope that the revisions to and reorganization of this section are agreeable to the reviewer.

**Reviewer #3: The Conclusions appeared adequate.**

--------------------

Editorial and Data Presentation Modifications?

Use this section for editorial suggestions as well as relatively minor modifications of existing data that would enhance clarity. If the only modifications needed are minor and/or editorial, you may wish to recommend “Minor Revision” or “Accept”.

**Reviewer #1: The paper needs a Figure that explains the different cohorts, substudies and numbers.**

We have added a study flow diagram that explains the different cohorts, substudies, and numbers.

**Fig 1 – the dotted lines for the 2SD threshold are not coming out clearly, they are indistinguishable from the mean difference line**

The original figure 1 was removed along with the content regarding biorepository specimens. However, the revised weight of the mean and 2SD threshold lines should now allow them to be distinguishable in Fig 3.

**line 601, pg 33: selected biomarkers**

Revised

**Reviewer #3: Dr. Arndt et al. present a report detailing multiple biomarkers of environmental enteric dysfunction (EED) and growth in a cohort of Malian children assessed for vaccine response. They make comparisons between the multiplex assay and more standard ELISA assays and compare to growth and vaccine response outcomes. This is an important topic relevant to the assessment of children in developing areas of the world.**

**I do have several concerns:**

**1. The authors in Tables 4 and 5 assess the relationship between seven different markers of EED (as quartiles of each marker) and seroconversion prevalence for Rotavirus IgA and IgG (Table 4) and seroconversion to Yellow Fever Virus or meningococcal A virus (Table 5), representing a total of 28 assessments. There are then an additional 56 analyses in Table 6 and 48 analyses in Table 7. There were p-values <0.05 for one of each of Rotavirus IgA, IgG and Yellow Fever Virus seroconversion in Tables 4 and 5 and for three additional analyses in Table 6 and 7 in Table 7. A few of these associations did exhibit very low p values. Nevertheless, several other associations had higher p-values closer to 0.05, as a reminder that the potential still remained that these associations could have happened by chance. With the number of assessments being performed, the authors should mention in their limitations section the potential for some spurious associations found because of multiple comparisons.**

We thank the author for this important critique and have added the following text to the limitations section:

Although numerous statistical tests are run and results presented in the study (e.g., Tables 3-6), no formal effort was made to control for the family-wise error rate such as the use of the Benjamini–Hochberg or Bonferroni method for alpha-correction. However, the use of unadjusted P-values is acceptable in exploratory analyses such as those in this study.

**2. In the Discussion (lines 627-637), the authors note the positive association between levels of IGF-1 and immune response to Yellow Fever Virus that was in the univariable model but not in the multivariable model. Among other factors, levels of IGF-1 are highly associated with advancing age, and the adjustment for age could have resulted in the amelioration of the univariable association. This possibility should be mentioned in the text.**

We appreciate the point raised by the reviewer and have added to following text to the discussion section:

Although IGF-1 concentrations are highly associated with age, within this cohort that was relatively homogenous with respect to age, IGF-1 and age did not appear to be correlated. However, because IGF-1 and baseline YF titer were negatively correlated (r=-0.1779), adjustment for baseline titer ameliorated the observed univariable association of IGF-1 and YFV seroconversion.

**Minor:**

**Line 481: In discussing the probability of rotavirus seroconversion based on sCD14 quartile, it appears that the p values for IgA and IgG have been reversed in the text.**

We appreciate the reviewer’s attention to detail, and have fixed the p-values in the text so they align with those in the supplemental table.

--------------------

Summary and General Comments

Use this section to provide overall comments, discuss strengths/weaknesses of the study, novelty, significance, general execution and scholarship. You may also include additional comments for the author, including concerns about dual publication, research ethics, or publication ethics. If requesting major revision, please articulate the new experiments that are needed.

**Reviewer #1: The manuscript by Arndt and colleagues focuses on environmental enteric dysfunction (EED), which is a highly prevalent but ill-understood disorder of low- and middle-income countries. Overall, they present an interesting set of analyses, focusing particularly on a new tool (MEEDAT) which evaluates a range of putative EED biomarkers in 10uL serum. This paper combines an evaluation of the MEEDAT performance with four distinct questions regarding EED and child health, growth and vaccine immunogenicity. As such, the manuscript is complex, too long, and hard to navigate since it loses focus with so many substudies. There is a need to substantially overhaul the manuscript to focus attention on one or two of these areas, because it is difficult for readers to take in all the information provided as it is currently presented.**

**The paper needs to be substantially simplified, since the cohorts, biomarkers and questions are of great relevance and interest, and the analyses generally sound, but it is currently un-navigable as a reader. I would suggest focusing on fewer questions, and limiting the interpretation to consistent findings that remain significant in multivariable analyses, then synthesizing the findings better for the reader.**

We appreciate the reviewer’s assessment of the manuscript. In response to this critique, we have substantially simplified the paper by removing content that emanated from the US-based biorepository specimens. We have reduced the overinterpretation of findings that are not significant in the multivariable analyses. We are hopeful that the removal of the associated tables and figures as well as the streamlining of study interpretation has improved the navigability and compensability of the manuscript.

Minor comments:

**1. Introduction lines 66-67 – update estimates based on 2018 data**

The estimates have been updated to reflect the 2018 Global Nutrition Report.

**2. A photograph of the MEEDAT system would be very helpful for readers unfamiliar with the Q Plex system**

We appreciate this reviewer feedback and have added **Figure 1** to the paper, which includes photograph(s) of MEEDAT array configuration and the Q-View™ Imager LS.

**3. Since this paper compares MEEDAT to ELISA, it is important to present quality control data on how well the ELISAs performed (e.g. intra- and inter-plate CVs for each assay).**

We appreciate this reviewer feedback, and have added intra- and inter-plate CVs for each assay to the paper (Lines 291-293 and Lines 303-305):

Each analyte within the validated MEEDAT array met the intra-assay and inter-assay specifications of <10% and <15% coefficient of variation (CV), respectively.

The standard ELISA kits exhibited intra- and inter-assay precisions of <15% and <10% CV, respectively as calculated from each assay run.

**4. Methods line 292 – what about implausible gains in height? If these were not also dropped from the dataset then it becomes biased**

This is an interesting point brought up by the reviewer. We note that only 1 child was removed from the growth analyses due to implausible height loss. More than 15 children (2%) gained between 5 and 7.7 cm (3 children gained >7 cm) during the 12 weeks of the study, with no obvious. Therefore, there is no clear rationale to categorize gains in height as implausible. The methods section has been updated to include the following:

Just one observation was dropped from growth analyses due to this implausibility criterion. No clear height gain outliers were observed with which to inform a similar rationale for removal.

**5. Table 8 – the comparison of children with (N=27) and without (N=27) rotavirus infection is underpowered and relies on a putative measure of rotavirus infection. The paper is stronger without this substudy.**

We thank the reviewer for this feedback.  Sample sizes were driven by sample availability from non-PRV group children who had a rise in anti-rota immunoglobin titer.  However, we have added the following text to the limitations section to address the sample size.

Case-control sample sizes were driven by sample availability from non-PRV recipients who had a rise in anti-rotavirus IgA or IgG over 28 days, and this sample size may not have provided adequate power to detect a statistically significant difference in baseline biomarker concentrations. Future work to answer this important question will need to come from a larger (preferably prospective) cohort of non-immunized children.

**Reviewer #2: It is a well written manuscript on a very important topic of public health research. The presentation of data can be improved by adding flow chart about study subjects. I struggled a lot to compare numbers and missing data, for eg. suppl. tables S3 and S4 do not match with number of biomarkers shown in main tables 4 & 5. Both method section and discussion are way too long, both needs to be concise and focused. In my view the presentation of results can be improved and words count can be decreased.**

**1- A general introduction of MEEDAT platform in a pictorial or a graphical abstract will help reader to understand it. I believe there are lot of anlaytes that can be tested but author can identify which analytes they are focusing for the current manuscript.**

We appreciate this reviewer feedback and have added **Figure 1** to the paper, which includes photograph(s) of MEEDAT array configuration and the Q-View™ Imager LS.

**2- It is important to add few sentences about field applicability of this assay. what type of lab infrastructure is needed to perform this test to assess the user friendly aspect of this test.**

We are grateful for this comment, as the absence of field applicability information for this assay was an oversight. The following text has been added to the discussion section:

Another strength of this study was the use of a technology suitable for deployment in low-resource setting laboratories to quantitate biomarkers in serum samples with MEEDAT. Use of the Quansys Q-View™ Imager LS requires a standard wet (e.g., microbiology) laboratory, minimal training of technicians familiar with running ELISA protocols, and a basic laptop computer for data processing and reports. The Imager LS platform is particularly well-suited for use in low-resource settings due to its comparatively low cost, few moving parts, small footprint, and protocol similarity to standard ELISA.

**3- It seems that there are two or three major objectives of this study. It would be nice if the objectives are clearly written in introduction section and results are aligned with those objectives.**

We appreciate this critical feedback from the reviewer and have added the following text to the end of the introduction:

The primary study objective was to test the laboratory performance of the novel multiplex assay by using it to quantitate plasma concentrations of IGF-1, FGF21, sCD14, I-FABP in child specimens and then compare with concentrations measured by monoplex ELISAs. The secondary objectives were to test if biomarkers of EED, systemic inflammation, and growth hormone resistance at baseline were associated with seroconversion to oral rotavirus vaccination rotavirus and 12-week height/weight growth. Finally, the exploratory objectives were to test if these biomarkers were associated with 1) seroconversion to parenteral meningococcus A or yellow fever vaccines and 2) natural rotavirus infection in children not given PRV.

**4- Regarding effect of EED biomarkers on YFV and MV, please give the rationale in introduction as they are not oral vaccine.**

We thank the reviewer for this feedback and have added the following text to the second paragraph of the introduction:

Although prior studies have observed relationships between immune response to orally administered vaccinations with EED and systemic inflammation biomarkers, no studies to date have tested whether biomarkers of GH resistance, systemic inflammation, or EED are associated with responses to parenteral vaccines or with risk of natural rotavirus infection. A priori, we expect that EED would not be associated with parenteral vaccine response due to the lack of mucosal immune system involvement.

**5-Baseline samples were shipped to Seattle for analyses. Rationale of collecting only baseline samples for EED study is not cleared to me, please mention if EED biomarkers were again measured at 28 day?**

Remnant baseline specimens were shipped to Seattle for analyses. Although remnant blood specimens were also available from day 28 of the parent study, EED biomarkers were not measured in these specimens due to the fact that our scientific question was to determine if concentrations of EED biomarkers measured at the time of vaccination were associated with subsequent immune responses. We did not hypothesize that the biomarker levels would change as a result of vaccination(s), nor did we hypothesize that the change in biomarker levels would be associated with these responses. While the day 28 specimens could perhaps have been used to test the stability of the baseline biomarker levels, limited financial resources and limited remnant sample availability kept us from considering these additional analyses.

**6-please specify if the biomarker data was previously published esp ELISA results?**

None of the biomarker data, including the ELISA results, have been published previously.

**7- Please discuss the difference in ages of bio repository and Mali study. What are the implications for pooling these results, if bio-markers are age specific?**

We have substantially simplified the paper by removing content that emanated from the US-based biorepository specimens for the reason mentioned in this comment, in addition to the difficulty expressed by several reviewers regarding difficulty with navigating the multiple cohorts and substudies included in the original manuscript.

Reviewer #3: (No Response)

--------------------

PLOS authors have the option to publish the peer review history of their article (what does this mean?). If published, this will include your full peer review and any attached files.

If you choose “no”, your identity will remain anonymous but your review may still be made public.

Do you want your identity to be public for this peer review? For information about this choice, including consent withdrawal, please see our Privacy Policy.

**Reviewer #1: No**

**Reviewer #2: Yes: Najeeha Talat Iqbal**

**Reviewer #3: No**
