# Supplementary material for: Validation of the Micronutrient and Environmental Enteric Dysfunction Assessment Tool and evaluation of biomarker risk factors for growth faltering and vaccine failure in young Malian children
Source: PLoS Negl Trop Dis. 2020 Sep 30;14(9):e0008711. doi: 10.1371/journal.pntd.0008711 (PMC7549819; doi:10.1371/journal.pntd.0008711)
Supplement: Attachment — Submitted filename: PNTD_MEEDAT_Review2_responses20200624.docx [file pntd.0008711.s011.docx]

# Authors’ responses to Reviewer Comments (post-revision)

The editors would like the authors to address the comments of reviewers 1 and 3 by including a few sentences highlighting the limitation of their study.

Reviewer's Responses to Questions

**Summary and General Comments**

Use this section to provide overall comments, discuss strengths/weaknesses of the study, novelty, significance, general execution and scholarship. You may also include additional comments for the author, including concerns about dual publication, research ethics, or publication ethics. If requesting major revision, please articulate the new experiments that are needed.

**Reviewer #1: The authors have addressed most of my original comments, included a flow chart which helps the reader to follow the different participants, and removed data from the US cohort. This generally helps to simplfy the manuscript.**

**I think the authors still place too much emphasis on their univariable results in their interpretation. For example, lines 575-576 of the discussion do not reflect the data shown in Table 5, where none of the adjusted RR of rotavirus vaccine seroconversion suggest an association with EED, whereas the conclusion states that EED may impair rotavirus vaccine responses in this older infant cohort. Similarly, lines 584 onwards still consider the IGF-1 findings to be important despite the fact that this association is no longer present in an adjusted model. I think this is an over-optimistic interpretation of the data, which largely shows no relationship between EED and vaccine responses.**
We appreciate the reviewer’s concern regarding the emphasis placed on univariable results in the discussion section. We feel that these unadjusted associations are still worth highlighting and warrant further investigation. We have added more clarifying language to the discussion section in order to emphasize to the reader that these were unadjusted associations:

Line 575: While previous data support the association between markers of EED and poor oral vaccine immunogenicity in young infants [12, 32], the univariable results in the present study suggests that EED may also be associated with poor oral vaccine immunogenicity in older infants (although such associations were not significant after adjustment).

Line 592: Accordingly, the significant crude relationship could potentially suggest that children who are producing higher levels of endogenous IGF-1 are more likely to receive protection from parenteral vaccines such as YFV.

Line 697: The significant associations between IGF-1 with YFV seroconversion and between I-FABP with rotavirus IgG seroconversion were only observed in unadjusted analyses; however, such findings warrant further investigation.

**There are two minor mistakes I think:

1. Line 467 seems to have an error in the 95%CI**
This error has been corrected (95% CI: 0.77, 1.00), thank you. **2. Line 33 of abstract - this is not a positive association. Higher AGP is associated with a lower relative risk of seroconversion**
Thank you for highlighting this error, the correction has been made in the abstract.
**Reviewer #2: all responses are adequately answered

Reviewer #3: The authors adequately addressed my concerns in their revision.**
